# The Best Material for Filling Pulp Canals

**Published:** 1903-05-15

**Authors:** 


### THE BEST MATERIAL FOR FILLING PULP CANALS.

This question has been discussed very vigorously by writers and speakers ever since the effort was first made to save pulpless teeth by filling the roots, and yet it is safe to say that there is no one material used to-day for this purpose which is in all respects satisfactory. Gutta-percha is probably more widely employed than any other, and it is has many desirable qualities which can not be ignored. The fact that in the hands of careful and painstaking operators the majority of pulpless teeth, the roots of which are filled with gutta-percha seldom give any subsequent trouble, would seem to be an endorsement of the material, but, after all, gutta-percha is not by any means ideal as a root filling.

In the excellent paper by Dr. A. E. Webster in the April issue of the *Dental Review* we see that gutta-percha is a very poor barrier to the entrance of bacteria, and if proof were wanting in this particular it is only necessary to remove old gutta-percha fillings which have been some time in the mouth, either from cavities of decay or from pulp canals, to be convinced that gutta-percha as ordinarily used is not impermeable. The odor of most of them will settle that. The contention may be made that much of the gutta-percha in use to-day is not of a good grade, which may account for the unsatisfactory condition in which these fillings are found, and yet it is the gutta-percha which is furnished us for this purpose and we must use it or none at all. It is feared that in the manufacture of cones for filling pulp-canals the best grade of gutta-percha is not always employed, and this particular question is well worth looking into by the profession.

One great advantage of gutta-percha is that it is a non-irritant and is therefore readily tolerated by the apical tissues. In this respect it is far preferable to oxychloride of zinc, which is sometimes used as a root-filling and which Dr. Webster found to be one of the best barriers to bacteria among the materials he tested. Oxychloride of zinc is a powerful irritant, and it is doubtful whether it can be safely used in canals having a large apical opening. In small or tortuous canals it is difficult or almost impossible to perfectly fill with any of the cements, and so the best combination we at present have for filling canals is probably to use a small short cone of gutta-percha at the apical end of the of the root to seal it and then fill the rest of the canal and the pulp chamber with oxychloride of zinc. By this arrangement several objects are accomplished. The oxychloride is kept from irritating the apical tissues while it effectually prevents the passage of bacteria from the mouth to the apical space. Being covered by the filling in the cavity proper it is not dissolved by the fluids of the mouth—a contingency to which it is susceptible when exposed. In very small canals some of the solutions of gutta-percha will probably answer the best purpose, covering this over with oxychloride of zinc in the pulp chamber.

But with any or all of the materials at our command the thing of prime importance is that the manipulative procedures of the operation be carried out with the utmost care. Experience has proved that if this is done the complaint in regard to materials will grow less.—Editorial in *Review*.

